# A Situational Overview of Prenatal Screening Services in Bhutan

**DOI:** 10.1002/puh2.70001

**Published:** 2024-08-27

**Authors:** Yeshey Dorjey, Tashi Gyeltshen, Thinley Dorji, Don Eliseo Lucero‐Prisno, Mimi Lhamu Mynak, Sonam Gyamtsho, Tashi Tshomo, Phurb Dorji

**Affiliations:** ^1^ Maternal and Fetal Medicine Unit, Department of Obstetrics and Gynaecology Phuentsholing General Hospital Phuntsholing Bhutan; ^2^ Department of Radiodiagnosis and Imaging Jigme Dorji Wangchuck National Referral Hospital Thimphu Bhutan; ^3^ Department of Internal Medicine Central Regional Referral Hospital Gelephu Bhutan; ^4^ Department of Global Health and Development London School of Hygiene and Tropical Medicine London UK; ^5^ Department of Paediatrics Jigme Dorji Wangchuck National Referral Hospital Thimphu Bhutan; ^6^ Office of the President National Medical Service Thimphu Bhutan; ^7^ Department of Obstetrics and Gynecology Jigme Dorji Wangchuck National Referral Hospital Thimphu Bhutan; ^8^ Khesar Gyalpo University of Medical Sciences of Bhutan Thimphu Bhutan; ^9^ Reproductive, Maternal and Newborn Health Unit Ministry of Health Thimphu Bhutan; ^10^ Maternal Fetal Medicine Unit Jigme Dorji Wangchuck National Referral Hospital Thimphu Bhutan; ^11^ Kidu Mobile Medical Unit His Majesty's Peoples Project Thimphu Bhutan

**Keywords:** amniocentesis, chorionic villi sampling, cordocentesis, noninvasive prenatal testing, prenatal diagnosis, prenatal screening

## Abstract

Prenatal genetic testing is to determine the possibility of the fetus having a genetic aberration or birth defect. Prenatal screening consists of serum analytes screening with or without nuchal translucency (NT) scanning or with cell‐free DNA (CfDNA) screening. Prenatal screening is recommended for all pregnant women regardless of the duration of pregnancy and maternal age or baseline risk. It is not advisable to screen with serum analytes and CfDNA concurrently to avoid discordant results. In developed countries, prenatal testing has been a part of routine antenatal care for a long time with adopting newer methods of screening and testing. In Bhutan, since the integration of the Safe Motherhood Program into primary healthcare in 1994, there has been an unprecedented improvement in obstetric care services. Almost all pregnant women attend antenatal and postnatal care, and 98.5% of deliveries are attended by trained health workers. The maternal mortality has reduced to 53 in 2023 from 770 per 100,000 live births in 1984 and the neonatal dealth has reduced to 15.2 per 1000 live births in 2023. However, despite improvements in the care of pregnant women, many babies are detected with congenital anomalies, syndromes, and birth defects during the postnatal period. Bhutan, being an underdeveloped country, could not initiate any form of prenatal testing program except for the anatomical scanning performed at 18–22 weeks of gestation. Early ultrasound dating scans, limited anomaly scanning, and growth scanning are offered to all pregnant women. There is a need to start centralized prenatal testing services in Bhutan to provide a comprehensive package of obstetric care to pregnant women. In addition, legal rights for parents to terminate severely deformed fetuses or severe genetic diseases before 24 weeks of pregnancy need to be established.

## Graphical abstract

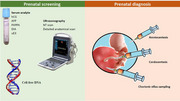

## 1 Introduction

Prenatal genetic testing started in the mid‐1970s with the introduction of the first serum analyte alpha‐fetoprotein (AFP) screening for neural tube defects. Since then, various screening concepts have developed, and prenatal screening has evolved to include ultrasound screening, serum analyte screening and the screening for fetal DNA [1]. Prenatal genetic testing is offered to women during pregnancy. This is to determine the possibility of the fetus having a genetic aberration or birth defect. Performing prenatal testing is useful in determining the options for special management during pregnancy and delivery to improve perinatal outcomes [2]. Pregnant women are screened with serum analytes β‐human chorionic gonadotropin (β‐hCG) along with pregnancy‐associated plasma protein A (PAPPA), AFP, and ultrasound scan for nuchal translucency (NT) in the first trimester (between 10 and 14 weeks of gestation); and quad screening involving four analytes, β‐hCG, AFP, dimeric inhibin A (DIA), and unconjugated estriol (uE3), performed in the second trimester (between 15‐ and 22‐week of gestation). Other prenatal screenings tests include combined first‐trimester and second‐trimester screening tests, integrated screening and serum integrated screening, sequential and contingent screening, and cell‐free DNA (CfDNA) [2]. Among the screening tests, CfDNA has the highest detection rate (99%) for Trisomy 21 as compared to first‐trimester screening (82%) and quad test (81%) [2]. A recommendation of one screening test for all patients is not suitable because individual prenatal test has its limitations. The Society of Obstetricians and Gynecologists of Canada (SOGC) and the American College of Obstetricians and Gynecologists (ACOG) recommend prenatal screening (serum analytes with or without NT ultrasound or CfDNA screening) and prenatal diagnostic testing (chorionic villus sampling [CVS] or amniocentesis) for chromosomal abnormalities for all pregnant women, regardless of maternal age or baseline risk [2, 3]. The patients should be provided one prenatal screening approach, performing multiple screening tests simultaneously should be avoided because it gives contradictory risk estimates [4].

Bhutan is a tiny landlocked Himalayan kingdom sandwiched between two giant countries, China in the north and India in the south. The population of Bhutan is 0.77 million with a land area of over 38,000 km^2^ and about 70% of the kingdom is covered with forest [5]. Since the introduction of modern medicines in the 1960s, healthcare services have been provided through three‐tiered health systems with Out‐Reach Clinics and Primary Health Centres at the primary level, and district hospitals at the secondary and referral hospitals at the tertiary levels [6]. Major reforms in maternal health took place after the introduction of the Safe Motherhood Program in 1994 [7]. Priority was given to the health sector by the Royal Government of Bhutan, and over the last four decades, maternal mortality has reduced to 53 in 2023 from 770 per 100,000 live births in 1984 [8, 9]. There is a drastic improvement in antenatal care (ANC) and postnatal care (PNC) visits, and 98.5% of deliveries were attained by trained health workers and achieved 98.0% of institutional deliveries [9]. As of now, almost all pregnant women are offered early ultrasound dating scanning and anomaly ultrasound scanning at 18–22 weeks of gestation. Pregnant women with fetal anomalies are referred to the district or tertiary hospital for further detailed anatomical ultrasound scanning and prenatal testing.

As of 2024, there is no prenatal screening program or guideline in Bhutan. There is a need for the initiation of appropriate, evidence‐based prenatal screening programs and develop guidelines for prenatal testing that will help in preventing the birth of an anomalous child. There is also a need to include prenatal screening as a part of routine ANC services. This article provides an overview of prenatal testing services for pregnant women in Bhutan and the challenges in providing such services in a resource‐limited setting.

## 2 State Policies on Healthcare Services in Bhutan

As of 2023, there are 53 hospitals, 183 Primary Health Centres,, and 554 Out‐Reach Clinics in Bhutan [9]. All aspects of healthcare services are provided for free by the Royal Government of Bhutan through all levels of care. Patients who require specific treatment that is not available in the country are referred abroad to empaneled hospitals for high‐end therapies at the expense of the state. Maternal and child health services, including ANC and PNC, are also provided for free through hospitals, Primary Health Centers, and Out‐Reach Clinics. The provision of free health care (inclusive of both modern and traditional medicine) is enshrined in the Constitution of Bhutan, Article 9, Section 21 [10]. Private health services are restricted to a few diagnostic laboratories and imaging studies in Bhutan.

## 3 Situation of Obstetrics Care in Bhutan

In Bhutan, the Reproductive Health (RH) Program started in 1994 with the launch and integration of the “Safe Motherhood Program” into the primary healthcare system [7]. Until such a time, there was no formal program to look after maternal and child healthcare. Thereafter, Bhutan has witnessed unprecedented progress in maternal and child healthcare services over the last three decades with support from the United Nations Population Fund (UNFPA), the United Nations International Children's Emergency Fund (UNICEF), and the World Health Organization (WHO) [7]. There was only 1 Bhutanese obstetrician in 1998; the number has increased to 22 obstetricians as of 2024, among which there are 3 maternal and fetal medicine specialists [9]. Comprehensive and basic emergency obstetric and newborn care EmONC centres were identified by the year 1999, and as of now, there are 10 comprehensive EmONC centres across the country with obstetricians who provide obstetric services [11].

The Royal Government of Bhutan accords high priority to the health sector with 22% of the current health expenditure allocated to the RH program that looks after obstetric care services [12]. Despite the improvement of obstetric care services, with only 22 obstetricians for the whole country, pregnant mothers particularly from far‐flung remote places face hurdles in accessing quality obstetric care.

## 4 Routine Obstetric Care Practices in Bhutan

Pregnant mothers are registered at the nearest Primary Health Centre or hospitals where, ANC and PNC follow‐up visits are conducted by trained health workers. Folic acid supplements are administered during the first trimester of pregnancy, and multi micronutrient, iron, calcium lactate, and vitamin C supplements are initiated in the second trimester and continued throughout pregnancy and postpartum period. In high‐risk pregnancies, aspirin is started at 12 weeks or before 16 weeks and continued up to 36 weeks of gestation. An early‐dating ultrasound scan is performed in the first and second trimesters, and a limited anomaly scan is performed at 18–22 weeks of gestation [13]. In Bhutan, all the ultrasound scanning is done by a sonographer. Sonographers are radiology technicians who have undergone short‐term training in basic obstetric ultrasound scanning and are not trained to do detailed anatomy scans. Radiologists in Bhutan do not provide advanced obstetric ultrasound scans.

Since 2009, the Reproductive Health Program recommends eight ANC visits for low‐risk pregnancies and a more frequent ANC follow‐up for high‐risk pregnancies [14]. At the Primary Health Centre, pregnant mothers are screened for diabetes, anemia, hypertension, and other chronic diseases. Delivery of low‐risk pregnancies is conducted in the Primary Health Centre, and high‐risk pregnancies are referred to the nearest comprehensive EmNOC centres equipped with obstetricians, pediatricians, and facilities for cesarean section [13].

## 5 Prenatal Screening Services

Prenatal genetic screening consists of different methods performed depending on the period of gestation. Prenatal screening with serum analytes (PAPPA, serum β‐hCG, and AFP), with or without NT scan, and quad screen (serum β‐hCG, AFP, DIA, and uE3) is performed during the first and second trimesters, respectively. There are other prenatal screening methods, namely, combined first‐trimester and second‐trimester screening tests, integrated screening and serum integrated screening, sequential and contingent screening, and CfDNA [2]. The detection rate for chromosomal disorders differs from one test to another, and among the screening tests, CfDNA has the highest sensitivity, specificity, and positive predictive value as compared with other tests [2]. However, none of the screening tests is perfectly suited for all patients; each of the screening tests has its limitations. Accurate timing with the duration of pregnancy and offering appropriate screening tests are important [2]. However, it must be noted that prenatal genetic screening is to determine the possibility of the fetus having a genetic condition or birth defect, and none of the tests is diagnostic of the condition.

In Bhutan, none of these prenatal screening tests are done because, currently, there is no cytogenetic laboratory to perform serum analyte testing and no geneticist to interpret the findings of genetic testing. However, in the case of some fetal anomalies detected while performing anomaly scanning at 18–22 weeks of gestation, those pregnant women are referred to the National Referral Hospital, Jigme Dorji Wangchuk National Referral Hospital, Thimphu, for detailed anatomical scanning. Here, blood samples are collected from pregnant women with high risk for genetic disorders (advanced maternal age, history of fetal anomaly, children with intellectual impairment, genetic disorders in the family, or multiple repeated miscarriages) [2], and the blood is sent to reference Lab in New Delhi, India, for cytogenetic analysis with CfDNA. The CfDNA report is available online after 2 weeks of receiving a sample in India. On the basis of the CfDNA report, couples are either advised to continue a pregnancy with serial ultrasound scanning or counseled for the need for prenatal diagnostic tests to confirm the diagnosis of the condition.

## 6 Prenatal Diagnostic Procedures

Ideally, screen‐positive pregnant women should undergo prenatal diagnostic procedures depending on the period of gestation and the indications [2]. The prenatal diagnostic procedures are CVS, amniocentesis, and cordocentesis. CVS is the procedure of obtaining a biopsy of chorionic villi (placenta), and it is performed between 10 and 13 weeks of gestation; amniocentesis involves drawing amniotic fluid from the amniotic sac, which is typically performed between 15 and 20 weeks; however, it can be done at any time later in gestation. Early amniocentesis can be performed from 10 to 14 weeks; however, this procedure has become obsolete and not recommended due to significant procedure‐related fetal complications [2]. Fetal blood sampling, also called cordocentesis, is performed from 18 to 20 weeks of gestation. Cordocentesis is rarely performed for genetic testing; however, it is done in conjunction with intrauterine fetal blood transfusion [15]. In Bhutan, because there is no laboratory facility to run prenatal diagnostic tests, prenatal diagnostic procedures are not done. A few cases were counseled to undertake such procedures in India and Thailand. It is high time for Bhutan to initiate and start performing prenatal diagnostic procedures.

## 7 Prenatal Diagnostic Tests

Prenatal diagnostic tests require fetal cells for testing, which are collected by prenatal diagnostic procedures (CVS, amniocentesis, or cordocentesis). The prenatal diagnostic tests include karyotyping or cytogenetic analysis, chromosomal microarray analysis (CMA), fluorescence in situ hybridization (FISH), targeted mutation testing, next‐generation sequencing (NGS), and whole exome sequencing (WES) [2, 15].

Karyotyping is the gold‐standard prenatal diagnostic test performed on a cultured fetal cell, and the result typically takes 2 weeks. It can assess all 23 pairs of chromosomes, including sex chromosomes. Karyotyping can detect chromosomal abnormalities that include trisomy, monosomy, deletions, duplications, translocations, inversions, and ring chromosomes.

CMA is performed on an uncultured fetal cell, and the turnaround time is faster, unlike karyotype. CMA is preferred to karyotyping and recommended in a setting of fetal structural abnormalities [16] because it can detect clinically significant chromosomal abnormalities in 6.5% of fetuses with normal karyotypes [17]. CMA can detect chromosomal abnormalities at the submicroscopic level throughout the human genome, which includes microdeletions and microduplications within a chromosome that are smaller and cannot be detected by conventional karyotype; they are referred to as copy number variants (CNVs) [15]. However, CMA cannot assess balanced chromosome translocations (which can be detected by karyotype), nor it can detect single‐gene disorders, which can cause various autosomal recessive/dominant and X‐linked conditions. Single‐gene disorders can be detected by targeted mutation analysis or by sequencing DNA (NGS or whole genome sequencing). Despite prenatal diagnostic tests being done a long time back in other countries and prenatal testing having evolved so much, Bhutan does not have a cytogeneticist and a cytogenetic laboratory to perform the diagnostic tests.

## 8 Fetal Anomalies and Birth Defects Detected at Birth

**FIGURE 1:**
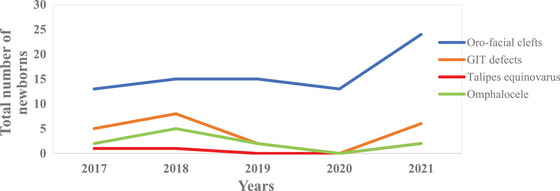
The types and the number of congenital anomalies detected in newborns in Bhutan (2017–2021).

Over the years, an increasing number of anomalies were detected at the time of delivery in newborn babies in Bhutan. Of these, oro‐facial clefts and gastrointestinal defects were the most common birth defects documented in the newborn as shown in Figure 1 [9].

Newborn babies were diagnosed to have congenital heart disease at birth, with atrial and ventricular septal defects being the most common disorders among heart diseases [18]. Many babies were diagnosed with the Down syndrome after delivery. More than 295 syndromic babies with disabilities inclusive of Down syndrome babies were taken care of by the Ability Bhutan Society, a nonprofit organization [19].

With the lack of routine prenatal genetic screening and prenatal diagnostic testing services in Bhutan, most syndromic or anomalous babies and congenital heart diseases are detected during the postnatal period. Over 1008–1344 miscarriages are reported across the health centres in Bhutan, and around 68 fetuses died during intrauterine life or were stillbirths [9]. More than 21 neonates per 1000 live births died during the first month of delivery [9], and another 15.1 infants per 1000 live births died during the first year of their lives [5, 9]. The miscarriage and intrauterine fetal demise or stillbirth are the consequences of genetic abnormalities and fetal disorders that have gone undiagnosed during the intrauterine period [20, 21]. Significant numbers of newborns are dying within the first year of their lives, which is partly attributed to undiagnosed fetal anomalies and intrauterine fetal infections. Had there been routine prenatal genetic screening, prenatal diagnostic testing, and invasive fetal therapy services in Bhutan, we would have had fewer miscarriages and intrauterine fetal demise and better perinatal outcomes.

## 9 Challenges for Prenatal Screening and Testing Services in Bhutan

Bhutan is a resource‐constrained country with a population of less than a million and major challenges in sustainable funding of the free healthcare system. Most of the health programs are donor‐dependent, including the Reproductive Health Program, which looks after the obstetric care services. With limited number of obstetricians in the country, the health system is not able to cater to all pregnant women with early routine prenatal screening with ultrasound scanning and other obstetric care. Early dating and limited anomaly scanning are performed and rely on the findings of the sonographers with detection rates dependent on the skills of the operator. Setting up prenatal screening and diagnostic testing services at the National Referral Hospital and training a cytogeneticist are associated with huge financial implications for the country. Privatization of health is not allowed by the existing policies [10], or else private companies could have established tertiary hospitals with prenatal diagnostic centres.

The Penal Code of Bhutan (2004), Chapter 11, Section 146, states that “abortion is illegal” except if it is done to save the life of the mother, when the pregnancy resulted from rape, incest, or when the mother is mentally unsound [22]. Thereby, the role of prenatal testing and diagnosis of genetic abnormalities will be limited to providing counseling to couples and helping them prepare to accept the anomalous or syndromic baby in their family. Medically, it is not justifiable to continue the pregnancy if the fetus has lethal anomalies, which are incompatible with life. In such cases, termination of pregnancy is required after thorough counseling of the couple, and obtaining informed written consent, and with formal legal support based on the Penal Code of Bhutan.

## 10 Recommendations on Prenatal Testing in Bhutan

In Bhutan, many pregnancies end up with miscarriages, intrauterine fetal demise, or stillbirth [9].

There is a need for the establishment of prenatal genetic screening and prenatal diagnostic testing centre at the National Referral Hospital to improve the overall pregnancy and perinatal outcomes.

After the centre is established, routine prenatal genetic screening and diagnostic testing options should be discussed and offered to all pregnant women regardless of age or risk of chromosomal abnormality. Screen‐positive results should not be used to make critical clinical decisions; rather, screen‐positive patients should undergo genetic counseling, a detailed anatomical ultrasound scan and should undergo prenatal diagnostic testing to confirm the diagnosis [2].

Prenatal screening should be a “single–time point screening approach” and use only one screening method. Screening with serum analyte and CfDNA should not be done concurrently, because seemingly discordant results can be more distressing to patients and will require unnecessary further testing.

Obstetric ultrasound scanning and ultrasonographic prenatal screening should be incorporated into the postgraduate residency training program, and all obstetric scanning should be performed by the obstetricians in the future. This will improve the detection rate of fetal abnormality and help in initiating early intrauterine fetal therapy, which can improve perinatal outcomes.

There is a need to train laboratory technologists in serum analyte and CfDNA testing and reporting. There is a need to train cytogeneticists and establish cytogenetics laboratories for performing prenatal diagnostic tests (karyotyping, CMA, FISH, targeted mutation testing, NGS, and WES).

The national health policy of Bhutan should incorporate prenatal screening services as part of the RH program. There is a need for the initiation of appropriate, evidence‐based prenatal screening programs or the development of national guidelines for prenatal testing to assist obstetricians in preventing the birth of an affected child. There is also a need to develop a plan, initiate a countrywide prenatal screening program, and include prenatal screening as a part of routine ANC.

There is a need to have a consultative meeting between multi‐stakeholders including lawmakers and re‐visit the section about abortion law in the Penal Code of Bhutan. Amendments of the penal code may include legalization of abortion and medical termination of pregnancies for lethal fetal anomalies, selective fetal reduction in the case of multiple pregnancies following in vitro fertilization and selective feticide in the case of a severe form of twin‐to‐twin transfusion syndrome (twin reversed arterial perfusion with acardiac co‐twin, anomalous co‐twin, and twin anemia polycythemia sequence).

## 11 Conclusion

In Bhutan, the proportion of pregnancies that end up with miscarriages, intrauterine fetal demise, or stillbirth and the proportion of newborns and infants die after delivery need a careful review. In addition there are a significant number of newborns detected with congenital syndromes after delivery, and many birth defects are detected at birth. There is an urgent need to establish prenatal screening and a prenatal diagnostic centre at the National Referral Hospital and adopt routine prenatal testing services during the antenatal period. To improve overall pregnancy and perinatal outcomes, a relook into health policies, multistakeholder engagement and adoption of adequate legal provisons are recommended.

## Author Contributions

Yeshey Dorjey conceived, conducted a literature search, and drafted the manuscript. Tashi Gyeltshen conceived, conducted a literature search, and reviewed the manuscript. Don Eliseo Lucero‐Prisno III and Thinley Dorji edited, reviewed the draft, and prepared the final. Sonam Gyamtsho, Phurb Dorji, Tashi Tshomo, and Mimi Lhamu Mynak edited and reviewed the draft. All authors reviewed the final draft for publication.

## Ethics Statement

The authors have nothing to report.

## Consent

The authors have nothing to report.

## Conflicts of Interest

Yeshey Dorjey is an Editorial Board member of Public Health Challenges and coauthor of this article. Thinley Dorji and Don Eliseo Lucero‐Prisno III are the Editor‐in‐Chief of Public Health Challenges and coauthors of this article. They were excluded from editorial decision‐making related to the acceptance of this article for publication in the journal.

## Data Availability

The data that support the findings of this study are available from the corresponding author upon reasonable request.
